# The Role of Vestibular Physical Therapy in Managing Persistent Postural-Perceptual Dizziness: A Systematic Review and Meta-Analysis

**DOI:** 10.3390/jcm14155524

**Published:** 2025-08-05

**Authors:** Diego Piatti, Sara De Angelis, Gianluca Paolocci, Andrea Minnetti, Leonardo Manzari, Daniel Hector Verdecchia, Iole Indovina, Marco Tramontano

**Affiliations:** 1 Laboratory of Neuromotor Physiology, IRCCS Santa Lucia Foundation, 00179 Rome, Italy; d.piatti@hsantalucia.it (D.P.); s.deangelis@hsantalucia.it (S.D.A.); g.paolocci@hsantalucia.it (G.P.); 2 Department of Biomedical and Dental Sciences and Morphofunctional Imaging, University of Messina, 98125 Messina, Italy; 3 Azienda Ospedaliera San Giovanni Addolorata, 00184 Rome, Italy; aminnetti@hsangiovanni.roma.it; 4 MSA ENT Academy Center, 03043 Cassino, Italy; lmanzari1962@gmail.com; 5 Department of Health Sciences, Universidad Nacional de La Matanza, B1754JEC Buenos Aires, Argentina; dverdecchia@unlam.edu.ar; 6 Department of Systems Medicine, Centre for Space BioMedicine, University of Rome Tor Vergata, 00133 Rome, Italy; 7 Department of Biomedical and Neuromotor Sciences (DIBINEM), Alma Mater University of Bologna, 40138 Bologna, Italy; marco.tramontano@unibo.it; 8 Unit of Occupational Medicine, IRCCS Azienda Ospedaliero-Universitaria di Bologna, 40138 Bologna, Italy

**Keywords:** vestibular physical therapy, vestibular rehabilitation, balance, persistent postural-perceptual dizziness, rehabilitation

## Abstract

**Background**: Persistent Postural-Perceptual Dizziness (PPPD) is a chronic vestibular disorder characterized by dizziness, instability, and visual hypersensitivity. Vestibular Physical Therapy (VPT) is commonly used, but its efficacy remains uncertain due to limited and heterogeneous evidence. **Objective**: This systematic review and meta-analysis aimed to evaluate the effectiveness of VPT in reducing dizziness and improving balance in individuals with PPPD. **Methods**: A systematic search of MEDLINE and PEDro was conducted in January 2025. Studies were selected following PRISMA guidelines and included if they assessed VPT interventions in patients diagnosed with PPPD. Risk of bias was assessed using the PEDro scale and the modified Newcastle–Ottawa Scale. The meta-analysis focused on pre- and post-intervention changes in Dizziness Handicap Inventory (DHI) scores using a random-effects model. **Results**: Six studies met the inclusion criteria. VPT significantly reduced DHI scores (pooled Hedges’ g = 1.60; 95% CI: 0.75–2.45), indicating a moderate to large improvement. Additional outcomes included improvements in postural control (e.g., mini-BESTest and posturography) and psychological well-being (anxiety and depression questionnaires). However, high heterogeneity (I^2^ = 92%) was present across studies. **Conclusions**: VPT may improve dizziness and balance in PPPD, though evidence is limited. Further high-quality trials with standardized protocols are needed.

## 1. Introduction

Persistent Postural-Perceptual Dizziness (PPPD) was defined in the International Classification of Vestibular Disorders in 2017 as a chronic vestibular disorder characterized by symptoms of dizziness, unsteadiness, or non-spinning vertigo occurring on most days for three months or more [1]. These symptoms are exacerbated by upright posture, active or passive motion, and exposure to visually complex or dynamic environments [1,2]. Accurate estimates of the prevalence and incidence of PPPD are not yet available due to the recent formalization of diagnostic criteria [3]. However, clinical epidemiological studies indicate that PPPD accounts for approximately 14% of dizziness-related consultations in general internal medicine [4], and up to 17% in a supra-regional specialized outpatient clinic at the German Center for Vertigo and Balance Disorders, LMU Munich, where it represents the leading cause of dizziness [5]. PPPD is frequently diagnosed in neurology and dizziness clinics, often in conjunction with other vestibular disorders [6]. Moreover, many individuals with chronic balance disorders previously classified under different diagnostic categories may now meet the criteria for PPPD [3]. At tertiary dizziness centers, phobic postural vertigo and chronic subjective dizziness, two conceptual precursors of PPPD, represent the second most frequent diagnosis, accounting for approximately 15–20% of all patient presentations [7]. These recent data underline the growing clinical relevance of PPPD; however, they also reflect the current limitations in establishing a definitive prevalence rate, as epidemiological studies are still relatively few and often constrained by heterogeneous diagnostic approaches. PPPD may be triggered by conditions causing vertigo, dizziness, unsteadiness, or balance dysfunction, including acute, episodic, or chronic vestibular syndromes [1,2]. Neurological disorders and psychological distress can also act as precipitating factors [1]. Neuroimaging studies suggest that PPPD is associated with altered activity and connectivity in the vestibular and visual cortices, hippocampus, and frontal lobes [8,9]. Unhelpful behavioral patterns often exacerbate psychological and functional symptoms, including fear of falling, anxiety, and depression [10].

Several approaches have been proposed to manage PPPD, including Cognitive Behavioral Therapy (CBT), Vestibular Physical Therapy (VPT), and pharmacological treatments [11]. For decades, VPT has been a cornerstone in the management of vestibular disorders. The classic VPT protocol, developed by Cawthorne and Cooksey in the mid-20th century, aims to promote vestibular recovery and compensation through exercises focused on gaze stabilization and balance training [12]. These exercises stimulate ocular and postural control systems, improve gaze stability, reduce dizziness and instability, and ultimately help restore quality of life [12,13]. Traditional VPT facilitates improvement via three main mechanisms: restoration, habituation, and adaptation [13,14]. These reflect the brain’s neuroplastic response to vestibular loss, involving synaptic remodeling (restoration), functional reorganization through existing or newly developed compensatory strategies (adaptation), and enhanced tolerance or suppression of disruptive stimuli (habituation). Currently, the evidence supporting non-pharmacological interventions for PPPD, such as VPT, CBT, and stress management, is limited [3]. Similarly, no strong evidence supports the efficacy of pharmacological treatments, including selective serotonin reuptake inhibitors (SSRIs) and serotonin–norepinephrine reuptake inhibitors (SNRIs) [3]. Several reviews have evaluated the effectiveness of both pharmacological and non-pharmacological interventions in PPPD [15], including a scoping review on the role of VPT [16]. These studies consistently highlight the need for further research. Given the increasing prevalence of PPPD [10], updating the current evidence base is essential.

The aim of this meta-analysis is to evaluate the effectiveness of VPT in the management of individuals with PPPD and to identify the most effective treatment strategies. In addition, reviewing the existing literature will help to identify future research directions and enhance understanding in this evolving field.

## 2. Materials and Methods

This systematic review was performed according to the PRISMA (Preferred Reporting Items for Systematic Reviews and Meta-Analyses) statement [17] and following the Cochrane Handbook for Systematic Reviews of Interventions [18]. The protocol was regularly approved and published in an international prospective register of systematic reviews (CRD42024582561).

### 2.1. Search Strategy and Eligibility Criteria

The electronic databases searched in January 2025 were MEDLINE (PubMed) and PEDro (Physiotherapy Evidence Database). The search terms used were (“persistent postural perceptual dizziness” AND “exercise” OR “treatment” OR “rehabilitation” OR “physical activity” OR “management”). The search terms were modified for each database, and appropriate subheadings were used for each database searched (for details, see Appendix A).

Controlled and non-controlled clinical trials (i.e., randomized or non-randomized trials), retrospective studies, case reports, case series, observational studies, reviews, and systematic reviews were included. No restrictions related to publication date, sex, and country were applied.

### 2.2. Study Selection and Data Collection Process

Duplicate records were identified and removed using the EndNOTE software (version 21.5). Two co-authors (SDA and DP) independently conducted the study eligibility assessment and data extraction process. In case of any disagreement, the opinion of a third author (MT) was used to reach an agreement. The first selection of studies was initially conducted based on the title and abstract, and afterwards, full-text articles were examined.

The summary of results was reported following the PRISMA (Preferred Reporting Items for Systematic Reviews and Meta-Analyses) statement [17]. The PRISMA checklist is available in the Supplementary Materials (Material S1).

Two authors (SDA and DP) independently extracted the following relevant features of the included studies: name of the primary author, publication year, participants, rehabilitative intervention, and outcome measures.

### 2.3. Risk of Bias

The methodological quality of evidence was assessed using the PEDro scale [19] for the controlled trials and using a modified version of the Newcastle–Ottawa Scale (NOS) for non-randomized controlled studies. The assessment was performed by two authors (SDA and DP); discrepancies were resolved by consensus with a third reviewer (MT) as an arbiter. The PEDro scale ranges from 0 to 10, and the modified NOS ranges from 0 to 7. In both scales, the maximum score shows a better methodological quality.

### 2.4. Meta-Analysis

A meta-analysis was conducted to evaluate the effectiveness of VPT on DHI scores, a widely recognized and validated patient-reported outcome measure that assesses the self-perceived impact of dizziness on physical, emotional, and functional aspects of daily life. The DHI was selected as the primary outcome due to its frequent use across the included studies. Only intervention groups treated with VPT were included in the analysis. Effect sizes were calculated using Hedges’ g, representing the standardized mean difference from pre- to post-intervention within each VPT-treated group. A positive Hedges’ g indicates a reduction in DHI scores, reflecting improvement. To account for within-subject pre-post correlations, a correlation coefficient (r) of 0.5 was assumed, following established conventions in the absence of study-specific values. A random-effects model was applied to account for expected between-study heterogeneity. The pooled effect size and 95% confidence intervals were computed accordingly. Between-study heterogeneity was quantified using the I2 statistic and Cochran’s Q test. Substantial heterogeneity was anticipated due to differences in study populations, intervention protocols, and durations.

### 2.5. PICO Question

The PICO model was used to develop the search strategy for the present systematic review [20]. The PICO tool was employed to effectively address and provide a structured response to our research question: “How effective is Vestibular Physical Therapy in managing individuals with Persistent Postural-Perceptual Dizziness in terms of reducing dizziness symptoms and improving balance?”.

## 3. Results

**Figure 1:**
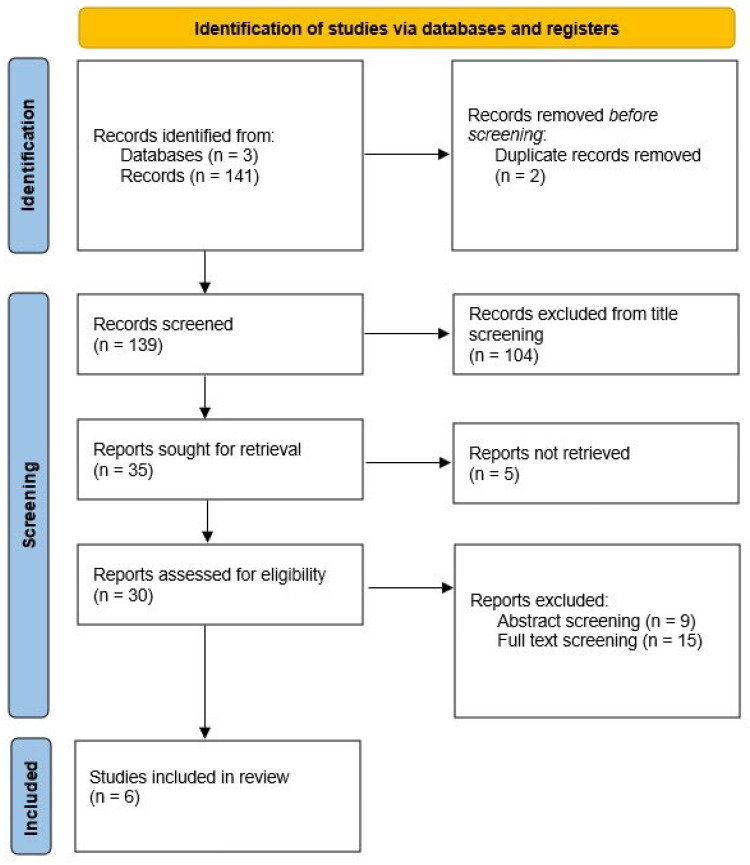
PRISMA flow diagram shoing the study selection process. Out of 141 records identified, 6 studies were ultimately included in the review.

The electronic search identified a total of 141 studies. After removing 2 duplicates, 139 articles remained eligible for initial screening. Titles and abstracts were reviewed based on predefined inclusion and exclusion criteria. Following title screening, 104 studies were excluded; an additional 9 studies were excluded after abstract screening. Five studies were excluded due to the unavailability of the full text, and fifteen studies were excluded following full-text review. Overall, 11 studies were excluded because they did not focus on VPT, as specified by Whitney SL [13], or they did not meet the Bárány Society diagnostic criteria for Persistent Postural-Perceptual Dizziness (PPPD) [1]. Furthermore, four reviews were excluded as they were not primarily focused on VPT [13]. Finally, six studies met all inclusion criteria and were included in this systematic review (Figure 1).

Table 1 presents a summary of included studies with their associated characteristics (name of the primary author, publication year, participants, rehabilitative intervention, comparator, outcomes, and outcome measures).

All the studies included in this review [21,22,23,24,25,26] emphasized the role of VPT in managing PPPD (Table 1). VPT may have been combined with other therapeutic approaches, particularly CBT or pharmacological interventions such as selective serotonin reuptake inhibitors (SSRIs); however, limited details were provided regarding patients’ concomitant pharmacological treatments or other therapies ongoing independently from the VPT sessions. This lack of detailed information restricts the ability to further analyze demographic or treatment-related characteristics and may introduce variability in baseline therapies that could potentially confound the observed outcomes. One study presented a new approach to managing VPT using virtual reality [21]. The Dizziness Handicap Inventory (DHI) and other questionnaires that gauge the subjective impression of balance are the main patient-reported outcome measures used. Additionally, tests are commonly used to assess PPPD-related variables such as depression (PHQ-9) and anxiety (GAD-7). Additionally, some researchers have made use of clinical scales like the mini-BESTest [23] or technology tools like Computerized Dynamic Posturography [21].

### 3.1. Risk of Bias

The RoB was assessed using the PEDro scale for Randomized Controlled Trials (RCTs). For non-RCT studies, the NOS was employed to evaluate methodological quality and derive an approximate risk of bias. The main sources of potential bias identified across the studies included: limited clarity and standardization of the interventions used, uncertainty regarding the accuracy and consistency of PPPD diagnoses, and challenges in maintaining participant blinding throughout the intervention period.

Table 2 shows the RoB of studies analyzed using the PEDro scale.

Table 3 shows the RoB of studies analyzed using NOS.

### 3.2. Results of Meta-Analysis of DHI

This meta-analysis synthesized data to evaluate the effect of VPT on DHI scores. The DHI was selected as the primary outcome measure due to its frequent utilization across the included studies, present in five out of six. Given the heterogeneity in study designs, which precluded a direct comparison of VPT versus a placebo or control intervention, the analysis focused on pre-to-post intervention changes within each study. Only the groups that were treated with VPT were considered in the meta-analysis.

**Figure 2:**
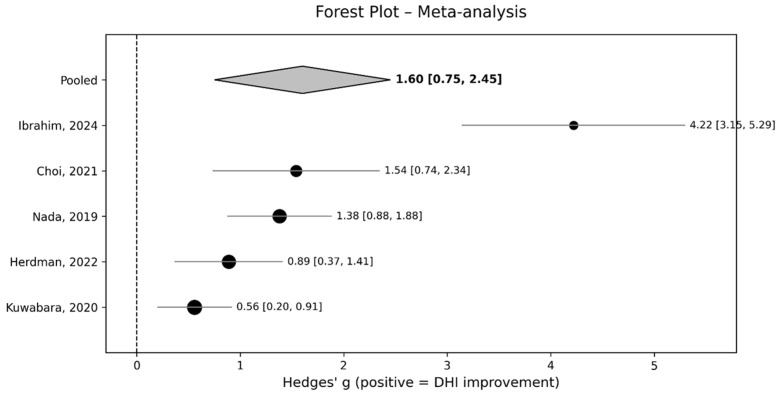
A forest plot showing individual and pooled effect sizes (Hedges’ g and 95% CI) for DHI improvement [21,22,24,25,26]. The studies are ordered in ascending effect size (Hedges’ g). The diamond indicates the overall effect (1.60 [0.75, 2.45]), favoring VPT intervention.

Hedges’ g was employed as the effect size metric to quantify the magnitude of change, with a positive value indicating a reduction in DHI scores. A random-effects model was utilized to account for the expected between-study heterogeneity. The pooled Hedges’ g for the meta-analysis was 1.601 (95% confidence interval [CI]: 0.752, 2.451), calculated with a correlation coefficient (r) of 0.5. The analysis revealed a statistically significant overall improvement following VPT. The between-study heterogeneity was substantial, with an I^2^ statistic of 92.0%. Individual study effect sizes ranged from 0.558 to 4.220. The Q test for homogeneity yielded a significant result (Q(4) = 44.26, value = 0.001), further supporting the presence of heterogeneity. Details of the effect sizes, confidence intervals, and study weights are provided in Table 4 and the forest plot (Figure 2).

### 3.3. Adverse Events and Safety

No adverse events were reported in any of the studies included in this review.

## 4. Discussion

The aim of this meta-analysis was to assess the efficacy of VPT in people with PPPD, a functional vestibular disorder characterized by chronic non-vertiginous dizziness, postural instability, and hypersensitivity to visual motion [1]. VPT is among the most frequently recommended interventions for PPPD, often in combination with CBT and pharmacological treatments [3,15]. In clinical practice, treatment approaches are frequently combined into a comprehensive, multidisciplinary management plan tailored to the individual patient’s symptoms and needs. This integrative strategy aims to address the multifactorial nature of PPPD, targeting both the vestibular symptoms and associated psychological factors such as anxiety and maladaptive behaviors. Despite its widespread clinical use, the evidence supporting VPT in PPPD remains limited and heterogeneous.

The therapeutic rationale for VPT traditionally relies on three key mechanisms: restoration of vestibular function, adaptation through sensorimotor substitution, and habituation to provocative stimuli [14]. These mechanisms have been well documented in peripheral vestibular pathologies, central vestibular dysfunction, and certain central nervous system disorders such as stroke [14,27]. However, their role in functional conditions such as PPPD remains unclear. In PPPD, the symptoms are not due to peripheral vestibular hypofunction, but rather to maladaptive multisensory integration, especially heightened visual dependence and impaired visuo-vestibular integration. This discrepancy raises important questions about how VPT protocols, originally developed for structural vestibular damage, translate into meaningful clinical benefits in patients with PPPD.

One of the major limitations observed across the included studies is the lack of detailed reporting regarding the specific content of the VPT protocols. While early approaches, such as Cawthorne–Cooksey exercises, provided a foundation for vestibular rehabilitation [28], contemporary models emphasize the importance of tailoring exercises to the individual’s functional deficits and symptom profile [14]. However, in the studies reviewed, such personalization was either insufficiently described or absent, making it difficult to discern which components of VPT may have been most effective, or for which subgroup of patients. It is therefore essential that all recent innovations and advances in VPT, such as individualized assessment tools and more tailored exercise, are incorporated both in future research and clinical practice. This integration would allow for more personalized and effective treatment approaches, ultimately improving patient outcomes.

The substantial heterogeneity observed in the meta-analysis (I^2^ = 92.0%) likely reflects these variations in treatment content, delivery, and patient characteristics. Moreover, in the context of PPPD, it is increasingly evident that traditional vestibular exercises, aimed primarily at promoting compensation for loss of peripheral input, may not fully address the pathophysiological mechanisms underpinning the disorder. Instead, interventions should target maladaptive visuo-vestibular interactions [29] and incorporate graded exposure to motion-rich environments. Approaches that integrate visual motion desensitization and strategies derived from CBT may provide more comprehensive symptom management and long-term benefit. Another important aspect to consider is the limited information available regarding concomitant pharmacological treatments. In PPPD, medications such as SSRIs are frequently prescribed as part of a multimodal therapeutic approach, often alongside VPT. The absence of consistent reporting on pharmacological interventions in the included studies represents a potential confounding factor, as it is unclear to what extent observed improvements can be attributed to VPT alone. Future studies should carefully document concurrent treatments to allow for more accurate interpretation of outcomes and to better isolate the effects of VPT.

It is also critical to consider the diagnostic limitations inherent to the current body of literature. Although we included only studies that reported PPPD diagnoses based on the Bárány Society criteria [1], the clinical diagnosis of PPPD remains challenging. The overlap with other vestibular and psychiatric conditions, and the absence of definitive biomarkers, raises the possibility of diagnostic misclassification. In several cases, more extensive vestibular testing, such as vestibular evoked myogenic potentials (VEMPs) [30] or video head impulse test (vHIT), might have revealed subclinical peripheral deficits or alternative etiologies for dizziness. This uncertainty introduces a risk of selection bias and limits the generalizability of the findings.

In summary, while our meta-analysis supports a significant overall improvement in DHI scores following VPT in patients with PPPD, the high degree of heterogeneity and methodological limitations of the included studies temper the strength of this conclusion. There is a need for more individualized VPT protocols, ideally developed and tested through controlled, prospective studies that directly compare conventional vestibular rehabilitation strategies with more modern, dynamic, and ecologically valid approaches aimed at enhancing visuo-vestibular interaction in defined patient cohorts.

## 5. Conclusions

VPT has shown effectiveness in alleviating symptoms in people with PPPD, but current evidence is constrained by significant heterogeneity across studies. Novel strategies targeting visuo-vestibular interactions are needed to enhance therapeutic specificity. High-quality RCTs comparing conventional and tailored approaches are crucial for informing clinical guidelines and enhancing rehabilitation programs.

## Figures and Tables

**Table 1:** Summary of clinical trials investigating VPT included in the review. The table includes study design, participants, intervention details, outcome measures, and main conclusions.

Authors, Year	Study Design	Participants	Intervention	Comparator	Outcomes	Conclusion
Choi, 2021 [21]	RCT	VPT+OS: 15 (8F) VPT: 13 (8F)	VPT with virtual reality, with or without optokinetic stimulation (OS), 20 min/week for 4 weeks	VPT using a virtual reality system	DHI, ADL, VVAS, Beck Anxiety Inventory, TUG test, Computerized Dynamic Posturography, Simulator Sickness Questionnaire	VPT group improved in all outcomes measures, where VPT+OS only improved in ADL and TUG.
Eldøen, 2021 [22]	Descriptive feasibility study	10 patients (1 dropout)	6-week web-based VPT program with daily exercises and educational content	NA	VSS, Niigata PPPD Questionnaire, PHQ-9, EQ VAS, semi-structured interview	Web-based VPT rehabilitation is a feasible and possibly effective treatment in PPPD patients.
Herdman, 2022 [23]	RCT	INVEST: 20 (16F) VPT: 20 (16F)	Six sessions of individual CBT-informed VPT (INVEST). Exercises were customized and focused on normalizing any maladaptive postural strategies early on and habituation	Standard VPT customized exercise program, performed in the clinic and at home, which included a range of general exercises (e.g., walking programs) and more specific adaptation, habituation, visual desensitization, static and dynamic balance exercises	DHI, VVAS, %TSI, EQ5D, B-IPQ, CBRQ, PHQ-9, GAD-7, PHQ-ADS, mini-BESTest	The CBT-informed VPT (INVEST) produced slightly better effects than traditional VPT, both in terms of average reduction in DHI and adherence to the treatment.
Ibrahim, 2024 [24]	Prospective study	30 patients	Clinic-based VPT (3×/week for 6 weeks) Home-based VPT: (15 min twice daily for 6 weeks: VOR X1 and walk with head movement exercises)	NA	HADS; DHI; SOT	VPT can reduce symptoms in patients with PPPD.
Kuwabara, 2020 [25]	Pilot study	27 patients	Combined ACT + VPT program. Patients were encouraged to conduct sets of nodding and head-shaking exercises (eyes open, eyes closed, fixating finger)	NA	DHI VSS HADS AAQ-II FFMQ	ACT combined with VPT for PPPD is a promising treatment with good feasibility.
Nada, 2019 [26]	RCT	VPT:30 (19 F) Control group: 30 (17 F)	VPT (gaze stability and walking exercises)	VPT+placebo integration	DHI	Customized home-based VPT suited to the patient’s aggravating factors is effective in PPPD management.

Abbreviations: RCT: Randomized Controlled Trial; DHI: Dizziness Handicap Inventory; ADL: Activities of Daily Living; OS = Optokinetic Stimulation; VVAS: Visual Vertigo Analog Scale; TUG test: Timed Up and Go Test; VSS: Vertigo Symptom Score; PHQ-9: Patient Health Questionnaire-9; EQ VAS: EuroQol Visual Analog Scale; CBT: Cognitive Behavioral Therapy; %TSI: Percentage of Time Symptoms Interfere with life; EQ5D: European Quality of Life-5 Dimensions; B-IPQ: Brief Illness Perception Questionnaire; CBRQ: Cognitive and Behavioral Responses to Symptoms Questionnaire; GAD-7: Generalized Anxiety Disorder-7; PHQ-ADS: Patient Health Questionnaire Anxiety and Depression Scale; mini-BESTest: Mini-Balance Evaluation Systems Test; SOT: Sensory Organization Test; ACT: Acceptance and Commitment Therapy; HADS: Hospital Anxiety and Depression Scale; AAQ-II: Acceptance and Action Questionnaire-II; FFMQ: Five Facet Mindfulness Questionnaire.

**Table 2:** PEDro scale score across 11 items: eligibility criteria, random allocation, concealed allocation, baseline comparability, blinding of subjects, blinding of therapists, blinding of assessors, adequate follow-up, intention-to-treat analysis, between-group comparisons, and reporting of point estimates and variability. Score excludes item 1. Green indicates “Yes” (criterion met), and red indicates “No” (criterion not met).

First Author, Year	1	2	3	4	5	6	7	8	9	10	11	Score
Nada, 2019 [26]	1	0	1	0	0	0	0	0	1	1	1	4
Choi, 2021 [21]	1	1	1	0	0	0	1	0	1	1	1	6
Herdman, 2022 [23]	1	1	1	1	0	0	1	1	1	1	1	8

**Table 3:** NOS score for non-RTC studies.

First Author, Year	Selection	Treatment Protocol	Outcomes	Score
Kuwabara, 2020 [25]	2	1	2	5
Eldøen, 2021 [22]	1	1	0	2
Ibrahim, 2023 [24]	1	0	1	2

**Table 4:** Data from the studies included in the meta-analysis.

Study	N of Participants	Mean Pre	SD Pre	Mean Post	SD Post
Choi, 2021 [21]	13	42	10	26	9.3
Ibrahim, 2024 [24]	33	70.5	12	23.2	9.4
Herdman, 2022 [23]	20	65.1	14.76	48.8	19.44
Nada, 2019 [26]	30	57.8	16.4	36	14.1
Kuwabara, 2020 [25]	35	49.1	20.4	38.5	15.9

## Data Availability

No new data were created for the present work.

## Supplementary Materials

The following supporting information can be downloaded at: https://www.mdpi.com/article/10.3390/jcm14155524/s1, Material S1: PRISMA 2020 Checklist.

## Abbreviations

The following abbreviations are used in this manuscript:

PPPD	Persistent Postural-Perceptual Dizziness
VPT	Vestibular Physical Therapy
DHI	Dizziness Handicap Inventory
VEMPs	Vestibular Evoked Myogenic Potentials
vHIT	Video Head Impulse Test

## Appendix A

Search Strategy in MEDLINE (PubMed): (“persistent postural perceptual dizziness” AND “exercis*” OR “treatment” OR “rehabilitation” OR “physical activity” OR “management”).

Search Strategy in PEDro: “persistent postural perceptual dizziness” AND “exercis*”.
